# Supplementary material for: Using a mixed opinion dynamics and innovation diffusion model to explore the ‘best game no one played’ phenomenon
Source: PLoS One. 2026 May 19;21(5):e0349217. doi: 10.1371/journal.pone.0349217 (PMC13186339; doi:10.1371/journal.pone.0349217)
Supplement: Attachment — Submitted filename: Referee_Report_PONE-D-26-01398.pdf [file pone.0349217.s005.pdf]

Referee Report on "Exploring the 'Best Game No One Played' Phenomenon Using A Mixed Opinion Dynamics and Innovation Diffusion Model"

Summary

This paper asks why certain innovative products receive favorable reviews yet fail to achieve widespread adoption—what the authors call the "best game no one played" phenomenon. To study this, the authors propose the BCAT model, which integrates two established modeling traditions: a bounded confidence opinion dynamics model (in the spirit of Deffuant et al. 2002), where agents update attitudes through pairwise interactions subject to a tolerance threshold, and an adoption threshold model of innovation diffusion (in the spirit of Watts 2004), where agents adopt a product only when a sufficient fraction of their neighbors have already done so.

The key modeling idea is that attitudes evolve continuously through social communication, but adoption is a discrete decision triggered by a threshold rule. An agent adopts only if (i) its attitude toward the product is positive (above a midpoint) and (ii) the proportion of its neighbors who have already adopted exceeds the agent's individual adoption threshold. The authors simulate this model on four canonical network structures—regular lattice, random, small-world, and scale-free—and conduct sensitivity analyses over five parameters: average adoption threshold, average attitude, standard deviations of each, and the bounded confidence parameter.

The main findings are that the average adoption threshold is the most influential determinant of adoption outcomes, followed by average attitude and bounded confidence. The paper also demonstrates that identical parameter configurations can yield vastly different adoption outcomes due to stochastic variation in initial conditions, illustrating how the divergence between favorable opinions and actual adoption can arise endogenously.

Overall Evaluation

The paper addresses an interesting question. The disconnect between opinion formation and adoption behavior is a well-recognized empirical phenomenon, and the idea of combining opinion dynamics with adoption threshold models to study it is natural and, to my knowledge, novel. The simulation results confirm the basic intuition that favorable attitudes are necessary but not sufficient for adoption, and provide useful comparative statics across network topologies.

My suggestions center on three themes. First, the theoretical framework could be presented more clearly, with greater emphasis on what makes the two models combine organically and where the key mechanisms diverge. Second, the most interesting aspect of the paper—the wedge between opinion and adoption—deserves deeper development, both through richer empirical grounding and through comparison with alternative explanations. Third, the paper would benefit from a discussion of how one might empirically distinguish the mechanisms proposed here from other theories that predict similar patterns. I elaborate below.

Major Comments

1. What Makes the Two Models Combine Organically?

The paper motivates the BCAT model as a combination of opinion dynamics and adoption thresholds, but the theoretical rationale for why these two components interact in the specific way they do could be articulated more sharply. In particular, the current presentation treats the two components somewhat modularly: agents first update opinions via bounded confidence, then check an adoption threshold. The reader is left to infer the interaction structure from the pseudo-code rather than from a conceptual discussion.

It would strengthen the paper to discuss explicitly: (a) what features of bounded confidence opinion dynamics—as opposed to, say, DeGroot-style averaging (Golub and Jackson 2010, 2012; Cheng et al. 2026) or voter model dynamics—make it a natural complement to threshold-based adoption, and (b) what structural properties of the combined model are not present in either component alone. For instance, bounded confidence naturally generates opinion clustering, and this clustering interacts with the adoption threshold in a non-trivial way: even if average opinion is favorable, clustered opinions can create pockets of agents whose local opinion environment never reaches the threshold for adoption. Making this mechanism explicit would give the reader a clearer sense of the model's theoretical contribution beyond the sum of its parts.

2. The Wedge Between Opinion and Adoption: What Are the Key Mechanisms?

To me, the most interesting aspect of the paper is the divergence between opinion dynamics and adoption behavior—the possibility that a population converges to favorable attitudes yet adoption remains low. The paper documents this phenomenon through simulation, but does not fully characterize the mechanisms that drive it.

From the current model, I can identify at least two distinct channels. The first is a *coordination failure* channel: even when all agents hold positive attitudes, adoption requires a critical mass of neighbors to have already adopted, and this critical mass may fail to materialize if early adopters are too dispersed across the network. The second is an *opinion clustering* channel: bounded confidence can partition the population into opinion clusters, some of which settle below the positive-attitude threshold, thereby mechanically reducing the pool of potential adopters regardless of network structure.

It would be valuable for the authors to disentangle these channels more carefully. For instance, one could ask: conditional on all agents having positive attitudes (eliminating the opinion clustering channel), how often does the coordination failure channel alone produce low adoption? Conversely, how much of the opinion–adoption gap is driven by opinion fragmentation versus threshold-based coordination failure? Separating these mechanisms would deepen the paper's theoretical contribution.

3. Empirical Grounding

The paper's motivating examples—the Palm handheld, a Taiwanese film, and service-oriented architecture—are mentioned briefly in the introduction but not revisited. Given that the opinion–adoption wedge is the paper's central phenomenon, the empirical case for its relevance deserves fuller development.

4. Alternative Theories and Distinguishing Predictions

The opinion–adoption gap is the paper's central puzzle, but other theories can also account for it: rational herding and information cascades (Banerjee 1992), where agents strategically delay rather than exchange opinions; network externalities and coordination games (Katz and Shapiro 1985), where adoption fails due to installed-base dependence independent of opinion dynamics; and global games with heterogeneous thresholds (Morris and Shin 2003), where higher-order belief failures prevent coordination even under favorable attitudes. Each mechanism implies different comparative statics—for instance, the BCAT model predicts a larger opinion–adoption gap in highly clustered networks, whereas the coordination games model predicts a larger gap in sparser networks. Discussing these distinctions, and suggesting how experiments or empirical tests might discriminate between them, would significantly enhance the paper's contribution.

Minor Comments

Reference Error: The Gong et al. (2020) reference contains what appears to be a corrupted title: "Hence, the source code used for this project may require significant modification to be applied to new studies."

References

[1] Banerjee, Abhijit V. "A simple model of herd behavior." *The quarterly journal of economics* 107.3 (1992): 797-817.

[2] Cheng, C., Han, X., Tong, X., Wu, Y., & Xing, Y. Degree-Weighted Social Learning. *American Economic Journal: Microeconomics*, 2026, forthcoming

[3] Golub, Benjamin, and Matthew O. Jackson. "Naïve Learning in Social Networks and the Wisdom of Crowds." *American Economic Journal: Microeconomics*, 2010, 2(1): 112–149.

[4] Golub, Benjamin, and Matthew O. Jackson. "How Homophily Affects the Speed of Learning and Best-Response Dynamics." *Quarterly Journal of Economics*, 2012, 127(3): 1287–1338.

[5] Katz, Michael L., and Carl Shapiro. "Network externalities, competition, and compatibility." *The American economic review* 75.3 (1985): 424-440.

[6] Morris, Stephen, and Hyun Song Shin. "Global games: Theory and applications." (2001).
